# Artificial Intelligence‐Enhanced Metasurfaces for Instantaneous Measurements of Dispersive Refractive Index

**DOI:** 10.1002/advs.202403143

**Published:** 2024-09-03

**Authors:** Trevon Badloe, Younghwan Yang, Seokho Lee, Dongmin Jeon, Jaeseung Youn, Dong Sung Kim, Junsuk Rho

**Affiliations:** ^1^ Graduate School of Artificial Intelligence Pohang University of Science and Technology (POSTECH) Pohang 37673 Republic of Korea; ^2^ Department of Electronics and Information Engineering Korea University Sejong 30019 Republic of Korea; ^3^ Department of Mechanical Engineering Pohang University of Science and Technology (POSTECH) Pohang 37673 Republic of Korea; ^4^ Department of Chemical Engineering Pohang University of Science and Technology (POSTECH) Pohang 37673 Republic of Korea; ^5^ Department of Electrical Engineering Pohang University of Science and Technology (POSTECH) Pohang 37673 Republic of Korea; ^6^ POSCO‐POSTECH‐RIST Convergence Research Center for Flat Optics and Metaphotonics Pohang 37673 Republic of Korea; ^7^ National Institute of Nanomaterials Technology (NINT) Pohang 37673 Republic of Korea

**Keywords:** biosensing, color filter, deep learning, glucose sensing, metasurface, Mie‐resonance, refractive index measurement

## Abstract

Measurements of the refractive index of liquids are in high demand in numerous fields such as agriculture, food and beverages, and medicine. However, conventional ellipsometric refractive index measurements are too expensive and labor‐intensive for consumer devices, while Abbe refractometry is limited to the measurement at a single wavelength. Here, a new approach is proposed using machine learning to unlock the potential of colorimetric metasurfaces for the real‐time measurement of the dispersive refractive index of liquids over the entire visible spectrum. The platform with a proof‐of‐concept experiment for measuring the concentration of glucose is further demonstrated, which holds a profound impact in non‐invasive medical sensing. High‐index‐dielectric metasurfaces are designed and fabricated, while their experimentally measured reflectance and reflected colors, through microscopy and a standard smartphone, are used to train deep‐learning models to provide measurements of the dispersive background refractive index with a resolution of ≈10^−4^, which is comparable to the known index as measured with ellipsometry. These results show the potential of enabling the unique optical properties of metasurfaces with machine learning to create a platform for the quick, simple, and high‐resolution measurement of the dispersive refractive index of liquids, without the need for highly specialized experts and optical procedures.

## Graphical abstract

Artificial intelligence is used to process spectral information from structural color metasurfaces to measure the dispersive refractive index of liquids. Images from a smartphone camera are demonstrated to measure the concentration of glucose in real time using a well‐trained neural network. The data‐driven system shows resolution comparable to conventional measurement techniques.

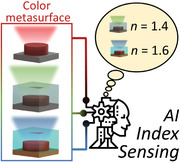

## 1 Introduction

The characterization of the optical properties of materials is an important process in numerous fields. The refractive index (*n*) is a fundamental characteristic that governs the optical response of a material such as the reflection, transmission, and absorption of thin films, the scattering of particles, and the confinement of light in metamaterials. Measuring the *n* of liquids can also be used to measure the concentration of biomolecules such as glucose.^[^ ^1^, ^2^ ^]^ This is usually achieved through a combination of complex measurements with specialist equipment. One such technique is ellipsometry, which requires both expensive equipment such as half‐wave plates, and compensators, as well as the manual input of a human expert to provide a suitable model to determine an accurate fitting of the measured Fresnel parameters.^[^ ^3^ ^]^ Another method of measuring *n*, Abbe refractometry, has been widely used by professionals in the food and agriculture industries due to its simplicity. However, it can only be used to measure *n* at a single wavelength, which limits the obtainable information from the target liquid or fruit. Using light‐matter interactions at the nanoscale, new techniques to measure *n* have been proposed by exploiting plasmonics,^[^ ^4^, ^5^ ^]^ as well as sharp high‐quality (Q) factor resonances,^[^ ^6^, ^7^ ^]^ and imaging.^[^ ^8^, ^9^, ^10^ ^]^

Metasurfaces, 2D arrays of subwavelength structures, have been developed over the past decade with numerous interesting photonic applications in various fields such as lensing and imaging,^[^ ^11^, ^12^, ^13^, ^14^ ^]^ holography,^[^ ^15^, ^16^ ^]^ and sensing.^[^ ^17^, ^18^, ^19^, ^20^ ^]^ Metasurface‐based refractive index sensors are typically designed to produce high‐Q resonances, and then *n* is inferred through spectral shifts or modulation of the linewidth in response to a change in the background index. These high‐Q resonances have been successfully realized using split‐ring resonators at microwave frequencies and bound states in the continuum (BIC).^[^ ^21^ ^]^ However, the resonance wavelength shifts are typically extremely small, on the order of pm, meaning that precise measurements must be undertaken within an extremely small bandwidth, while the experimental fabrication of BICs additionally requires highly accurate nanofabrication techniques. Therefore, there is a large void to be exploited using new strategies for the quick, simple, and high‐resolution measurement of *n* that could be solved using easy‐to‐fabricate metasurfaces alongside the powerful abilities of artificial intelligence (AI).

In the field of nanophotonics, machine learning and AI has been employed in various ways, from the inverse design of metasurfaces,^[^ ^22^ ^]^ to the post‐processing of optical data.^[^ ^23^ ^]^ Notable examples include expanding the limits of optical data storage,^[^ ^24^ ^]^ adaptive self‐cloaking,^[^ ^25^ ^]^ and image processing.,^[^ ^26^ ^]^ while recently, machine learning‐powered ellipsometry has also been demonstrated.^[^ ^27^ ^]^ By including AI in the system, the requirement for a human expert to fit the data was alleviated for the automatic characterization of the optical properties of films. Colorimetric sensors have found use for smell detection,^[^ ^28^ ^]^ while their inverse design has been proposed to determine the concentration of a solution.^[^ ^29^ ^]^ Furthermore, deep learning has been used to differentiate viruses.^[^ ^30^ ^]^ and to classify contaminants.^[^ ^31^ ^]^ based on their refractive index.

**Figure 1:**
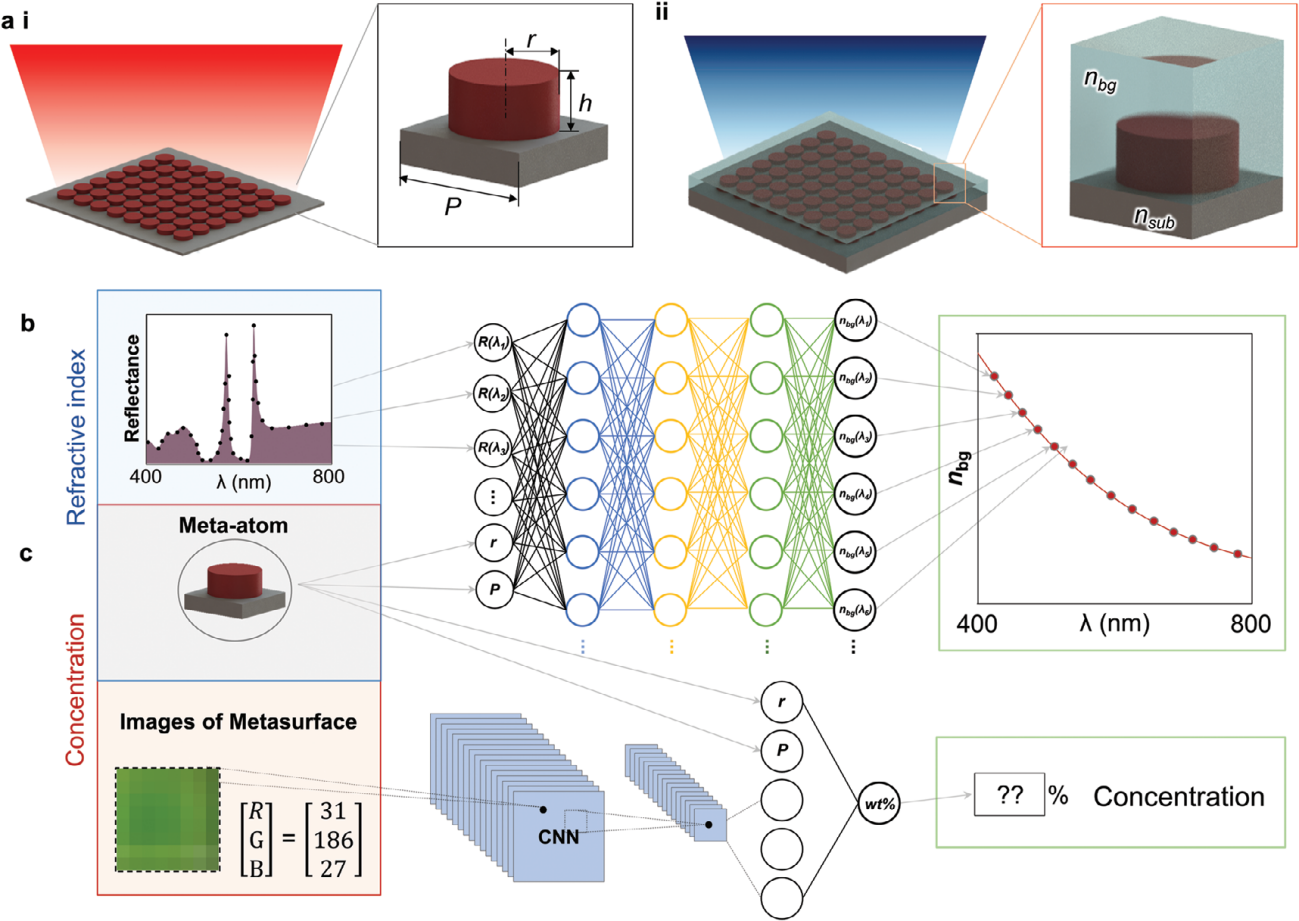
Concept of the proposed refractive index (*n*) measurement system by enhancing metasurfaces with machine learning. (a) The reflected color of a metasurface is defined by the geometric parameters of the unit cell, such as the radius (*r*), periodicity (*P*), height (*h*), and *n* of the material, background (*n*_bg_), and substrate (*n*_sub_). Therefore, different colors are reflected when the metasurface is in a background of (i) air, and (ii) covered with *n*_bg_. (b) Detecting *n*_bg_(*𝝀*) by enhancing metasurfaces with AI. The input data includes geometric parameters of the fabricated metasurfaces, as well as the measured reflectance or RGB values from a captured image. The DNN takes these parameters and provides an instantaneous measurement of the dispersive *n*_bg_(*𝝀*) or (c) the concentration of a solution.

Here, we design and experimentally demonstrate a robust refractive index sensing system for instantaneous measurements of the dispersive *n* over the whole visible regime using metasurfaces and machine learning. The reflected color of the metasurface is highly influenced by the *n* of the surrounding background medium (*n*_bg_) (**Figure** **1**a), which generally possesses a wavelength‐dependent dispersion, i.e.*, n*_bg_ *(𝝀)*. With a well‐trained deep neural network (DNN) and a single measurement of the reflectance from an arbitrarily chosen metasurface that produces structural color, we successfully demonstrate instantaneous measurements of *n*_bg_ *(𝝀)* of various liquids (Figure 1b). Furthermore, we prove the ability of the DNN to retrieve *n*_bg_ *(𝝀)* using any standard charge‐coupled device  (CCD), specifically first from an optical microscope, then from a smartphone camera. As a proof‐of‐concept application, we develop a real‐time *n* measurement system by combining the metasurfaces with a microfluidic channel, highlighting the potential uses for this integrated machine learning and metasurface platform for applications in various fields, using everyday equipment. Furthermore, despite causing a barely indistinguishable color change to the metasurfaces, we employ this simple setup to successfully detect the concentration of glucose dissolved in water. Compared to conventional *n* measurement methods, the resolution of our system is measured to be ≈10^−4^, which is comparable to commercial Abbe refractometers and ellipsometry.^[^ ^32^ ^]^ The required hardware consists only of a conventional CCD and a light source, which results in a cheap and extremely compact setup. The time taken for each measurement is almost instantaneous after the DNN has been trained and requires no additional input from a human expert which means the system can be easily implemented and used by anyone without the need for specialist training. A comparison of our proposed platform with other methods is provided in Note S1 (Supporting Information).

## 2 Results and Discussion

There have been numerous demonstrations of structural color metasurfaces using various different materials, from plasmonics using metals,^[^ ^33^ ^]^ to all‐dielectric platforms.^[^ ^34^, ^35^ ^]^ The reflection spectra of metasurfaces can be controlled through the makeup of the meta‐atoms, specifically the material and geometric parameters, as well as the *n* of the substrate (*n*_sub_) and *n*_bg_.^[^ ^36^, ^37^ ^]^ Here, we employ low‐loss amorphous silicon (a‐Si:H).^[^ ^38^ ^]^ meta‐atoms on a silica (SiO_2_) substrate as the materials for our metasurfaces, however, any other dielectric material could be substituted as long as it can produce structural color. Based on previous research, we select a meta‐atom height (*h*) of 110 nm and periodicities (*P*) from 200 to 400 nm, and radii (*r*) of 0.26*P*–0.36*P* to produce a large palette of reflected colors and intensities (Note S2, Supporting Information). By changing *n*_bg_, the reflectance of the structural color metasurfaces is modulated over the whole visible region in a non‐intuitive way as the hybridized resonances of the scattering multipoles and lattice resonances are simultaneously modified (Note S3, Supporting Information).^[^ ^39^ ^]^ As we do not employ high‐Q resonances here, there is no 1‐to‐1 mapping between the sharp spectral response and a change in *n*_bg_. Nevertheless, machine learning is a powerful tool that can uncover the complex hidden relationships and correlations in data. Therefore, we design and train DNNs that can instantaneously provide *n*_bg_ *(𝝀)* at visible wavelengths given the geometric parameters of a metasurface (i.e.*, P* and *r*) along with additional information related to the reflected color.

### 2.1 Measurement of *n*_bg_ *(𝝀)* from Reflectance Spectra

**Figure 2:**
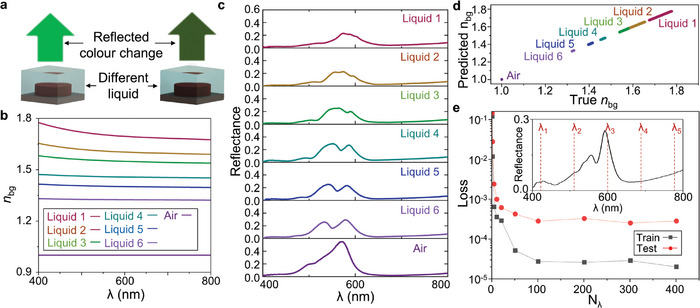
Experimental results of the measurement of refractive index from reflectance spectra. a) Changing the background index (*n*_bg_ *(𝝀)*) that surrounds the meta‐atoms, the reflected color is changed. b) The dispersive *n(𝝀)* of the 6 liquids used in this work, as well as air. c) Experimentally measured reflectance spectra for a metasurface coated with each liquid. d) True versus predicted *n*_bg_ *(𝝀)* from the unseen test data after training. e) Resolution of the trained model for a given number of probed wavelengths (N*_𝝀_*). The inset shows a schematic of probing 5 wavelengths across the visible spectrum (400–800 nm). Grey squares: training data; Red circles: test data.

In order to collect data to train our DNN, we fabricated and measured 24 individual structural color metasurfaces. When the metasurfaces are coated with a liquid, the change in *n*_bg_ *(𝝀*) results in a visible change of color (**Figure** **2**a). Each metasurface was made up of a 200×200 𝜇m^2^ array of meta‐atoms with 6 different values of *P* (from 200–400 nm) and 4 values of *r* (from 0.26*P*–0.32*P*). We measured the reflectance using a homemade setup under a white light source (Xenon‐lamp, Newport) with a spectrometer (HORIBA, iHR320) for the metasurfaces in air and coated with 1 of 6 different liquids (Cargille, refractive index liquids) with a known *n*(*𝝀*) (Figure 2b). The measured spectra for the metasurface with *P* = 280 nm and *r* = 0.32*P*, along with *n*_bg_ *(𝝀)* for each liquid are shown in Figure 2c. The measured spectra of the remaining metasurfaces are provided in Note S4 (Supporting Information). It is clear to see that the Fano‐type reflection peak in air is modulated when *n*_bg_ is changed using the coated liquid. To avoid any erroneous measurements, we perform each spectral measurement 5 times, coating and cleaning the metasurfaces each time. An example of the 5 measurements for one of the metasurfaces is provided in Note S5 (Supporting Information). This has the added benefit of providing us with more data for training the DNN model, while also helping the model to learn with somewhat noisy data without having to employ data augmentation techniques to introduce artificial noise. We therefore utilized a total dataset of 1176 measurements. We used a 90:10 training to test data split, resulting in a training dataset of 1058, with the remaining 118 for testing. The specifications of the DNN are provided in the Methods section. After training, the unseen test data was used as input to the trained model, and *n*_bg_ *(𝝀)* was successfully obtained for all of the different refractive index liquids (Figure 2d). All values of the dispersive *n*_bg_ *(𝝀)* were predicted by the model with a mean loss of 2.8 × 10^−4^. This reaches the original resolution of ≈10^−4^ for the refractive index liquids (Figure 2e). As each spectral measurement originally consists of 7143 data points over the visible spectrum (𝝀 *=* 400–800 nm), we investigated the effect of pre‐processing the data by interpolating each measurement down to a given number of points (*N_𝝀_*) across the visible spectrum (Figure 2e). The resolution of the predicted *n*_bg_ *(𝝀)* reaches ≈1.0 × 10^−3^ when *N_𝝀_* is 11 and decreases until *N_𝝀_* reaches the 100s where the resolution is ≈3.0 × 10^−4^. This is reaching the limitations of the resolution of the known values of the refractive index of the liquids, highlighting the potential of our system for high‐resolution measurements of *n*_bg_ *(𝝀)*.

### 2.2 Measurement of *n*_bg_(𝝀) from Reflected Color Images

**Figure 3:**
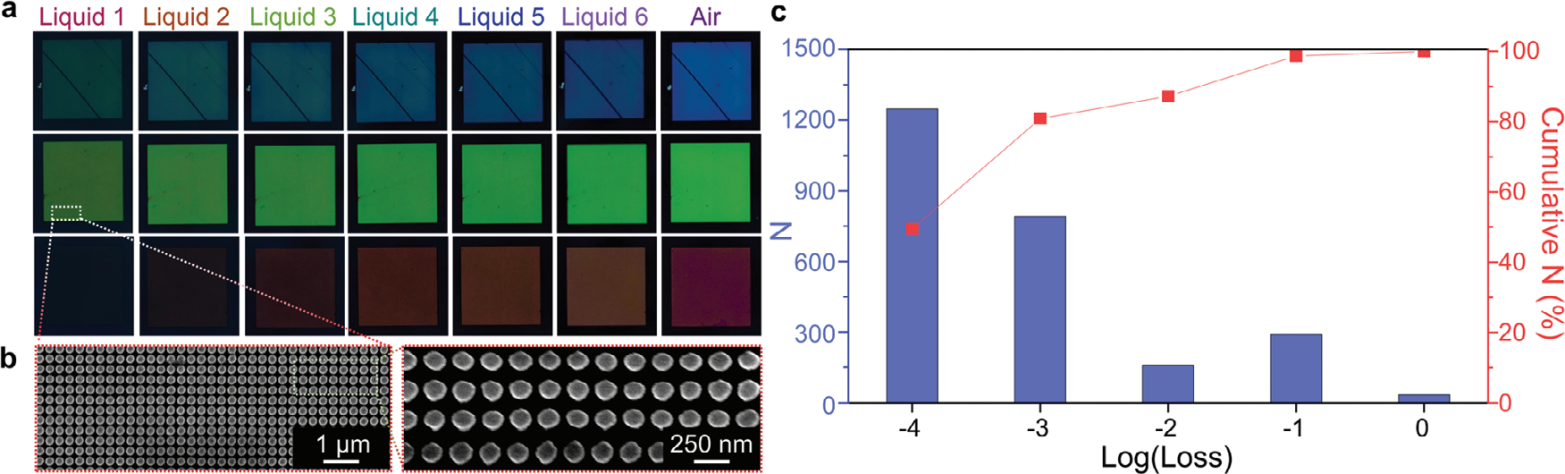
Experimental results of the measurement of refractive index from the RGB channels of a microscopy image. a) Optical microscope images of the 200 × 200 𝜇m^2^ metasurfaces that reflect red, green, and blue colors in air and coated with the 6 liquids. b) Scanning electron microscope images of the green metasurface. c) Accuracy of the *n*_bg_ *(𝝀*) measurement of the unseen test dataset using the RGB channels from the microscopy images.

When using spectral information as the input data to the DNN, the measurement still requires a form of specialist equipment in the form of a spectrometer. However, since we use metasurfaces that reflect structural colors, we next explore the measurement of *n*_bg_ *(𝝀)* using the red, green, and blue (RGB) channels of a CCD. We prove that as long as the CCD and source light are consistent between the data used to train the model and the wanted measurements, any combination of CCD and input light could be used. Therefore, measurements of *n*_bg_ *(𝝀)* across the visible spectrum with any kind of CCD would be possible, in an extremely easy, quick, and cost‐effective manner. First, we use a well‐defined and consistent experimental setup with an optical microscope. Images of the metasurfaces in the background of air, as well as coated with the same liquids are captured using the CCD. The results of the metasurfaces with *P* = 200, 280, 400 nm and *r* = 0.32*P* are shown in **Figure** **3**a, as well as scanning electron microscopy images (Figure 3b). These metasurfaces are chosen as they represent the brightest red, green, and blue colors, while the rest of the results can be found in Note S6 (Supporting Information). It should be noted that the lines and discrepancies that can be seen on the metasurfaces occurred after fabrication due to mishandling during experimentation; however, it has no adverse effect on the results. On the contrary, it further highlights the robustness of our platform using the integration of metasurfaces and AI for the measurement of *n*_bg_ *(𝝀)*. The data was prepared by cropping 8 × 8‐pixel samples from the complete images. Since the captured color of the metasurfaces is not perfectly consistent across the whole image, which can be attributed to local fluctuations in noise in the CCD, incident light, or physical defects from the nanofabrication of the metasurfaces, a form of physical data augmentation in inherently achieved. We prepare a total of 25 200 images, with a train‐to‐test split of 90:10, giving us a dataset that consists of 22 680 training data and 2520 unseen test data. Since the input now consists of 8 × 8 pixel images, we modify the DNN to include convolution layers, which have been proven to be well‐adapted for multichannel image data.^[^ ^40^, ^41^ ^]^ After training, the DNN has successfully learned the relationship between the input images and geometric parameters of the meta‐atoms and *n*_bg_ *(𝝀*) of the coated liquid. The mean loss of the fully trained DNN over the unseen test dataset is 9.7 × 10^−3^. 80% of the predicted *n*_bg_ *(𝝀*) lies within 10^−3^ of the true values, with 49% within the original resolution of the dataset of ≈10^−4^ (Figure 3c). With this, we have proven the potential for this AI‐enhanced metasurface system to measure *n*_bg_ *(𝝀*) instantaneously and accurately across the whole visible regime, using a simple color image.

### 2.3 Real‐Time Measurement of Glucose Concentration

**Figure 4:**
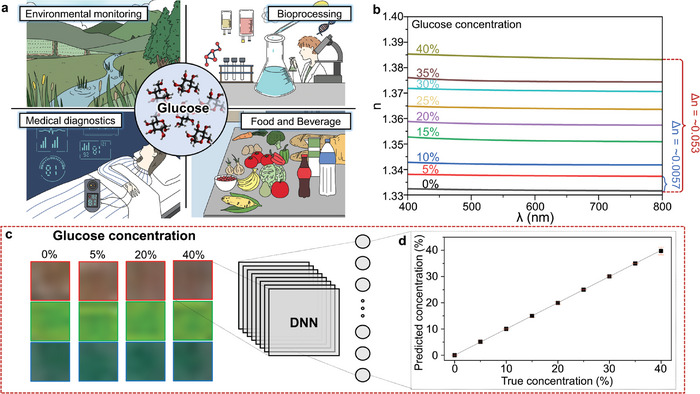
Application of glucose concentration sensing system with the AI‐enhanced metasurfaces. a) Schematic illustration of the potential uses for optical glucose sensing. b) Calculated *n* of different concentrations of glucose in water. c) Cropped images of the metasurfaces taken from videos using a smartphone, infiltrated with 0%, 5%, 20%, and 40% glucose concentration solutions. Using this data as the input to the DNN, we are able to d) measure the concentration of glucose in the solution. Error bars denote the standard deviation.

We finally confirm the suitability of our platform in real‐world situations by employing our simple, cheap, and easy‐to‐use setup to determine the concentration of glucose dissolved in water. Optical glucose sensing has numerous potential applications in various fields (**Figure** **4**a),^[^ ^42^ ^]^ such as monitoring glucose levels in soil and water to give information about the health of the natural environment, in bioreactors during the production of vaccines and pharmaceuticals, in medical diagnostics for non‐invasive sensing, as well as in the food and beverage industry to provide vital information on the quality of food and the existence of potential contamination. We note that the all‐dielectric materials used in our metasurface (namely, a‐Si:H and SiO_2_) are non‐toxic, so could be potentially implemented in all of the above applications without any negative side effects on human beings.

We prepare the microfluidic channel with different concentrations of glucose dissolved in water and record the change of color reflected from the metasurfaces. Although we proved that *n*_bg_ *(𝝀)* can be measured using a well‐trained DNN alongside metasurfaces using an optical microscope setup, this setup has obvious limitations in that it still requires a form of specialist equipment, which is not only bulky, but is also not readily available outside of the lab environment. Therefore, we design a proof‐of‐concept experiment using a microfluidic channel to actively inject and remove liquids across 400 × 400 𝜇m^2^ metasurfaces that reflect red, green, and blue with *P* = 400, 280, and 200 nm respectively, each with *r* = 0.32*P*. A standard smartphone is used to capture a video of the metasurfaces in real time. Care was taken to ensure that the camera settings, such as the aperture, exposure, and ISO were constant throughout the experiments. Details can be found in the Methods section. This simple, easy‐to‐setup, and user‐friendly platform provides a way for instantaneous measurements of the dispersive refractive index without the need for any specialist equipment, knowledge of optics, or expert training. Images of the experimental setup are shown in Note S7 (Supporting Information). The different concentrations of glucose are pumped through a microfluidic channel from the reservoir, and infiltrate the metasurfaces, An LED source is fitted to an optical rail to illuminate the metasurfaces with a constant source. Note that this is a different source from the previous experiments using traditional microscopy, and any consistent white light could be chosen. The liquids take ≈8 s to fully cover the metasurfaces and a similar amount of time to be cleaned by injecting distilled water after each use. Videos of the liquids infiltrating the metasurfaces are provided in Video S1 (Supporting Information). Cropped color swatches of the metasurfaces when covered by the glucose solution are shown in Figure 4c. The red, green, and blue colors are reflected and their modulations due to the refractive index liquids are noticeable in the zoomed‐in color swatches.

The *n* of the solution depends on the concentration of glucose, which when coated, will change *n*_bg_ *(𝝀)* and in turn the color reflected by the metasurface. Therefore, by recording the RGB values of the reflected images and training a DNN, we aim to measure the glucose concentration with an extremely simple and cheap method that could be undertaken without any expert training. We calculate the refractive index of the glucose solution at concentrations between 0% and 40% in steps of 5% using data from the literature (Figure 4b).^[^ ^43^ ^]^ The difference of *n* from 0% to 40% concentration is fairly small (≈5.3 × 10^−2^), which means that the changes in the reflected color will also be negligible to the human eye. However, enabling the microfluidic channel‐integrated metasurfaces with AI allows us to distinguish even such tiny modulations in color.

We use the frames of the videos and the metasurface geometric parameters as the input data to a DNN which performs regression to output a single continuous value of the concentration of glucose as the output. A total number of 5400 images were collected and a 90:10 train to test split was again employed, giving 4860 training data and 540 unseen test data. After training the DNN, despite the extremely small difference in *n*_bg_ *(𝝀)* that occurs due to the increase of glucose concentration (Figure 4b), and the correspondingly minor change in the reflected color (Figure 4c), we are able to successfully measure the concentration of glucose with only the RGB value information from a smartphone camera (Figure 4d). The mean loss of the unseen test dataset is 0.11, corresponding to a fraction of a percent difference compared to the true value, for measurements of the concentrations of glucose from 0% to 40%. Qualitatively, the color difference (ΔE, CIEDE2000) between 0% and 40% concentration images is 0.05, 0.04, and 0.01 for the red, green, and blue metasurfaces, respectively. This corresponds to ˂0.1× the minimal just noticeable difference of 0.5, highlighting the tiny change in color that the DNN is able to differentiate between. The inference time of the DNN is in the order of milliseconds, so could be integrated into a smartphone application or lightweight program that is processed on a device for real‐time measurements.

## 3 Conclusion

In conclusion, we have successfully demonstrated an integrated nanophotonics and AI platform to measure *n(𝝀)* of liquids. After proving the potential of the concept using full spectral measurements of the reflectance of metasurfaces, we further validated the performance using different light sources and sensors, through microscopy and the CCD of a conventional smartphone with an external LED light. The use of color to measure *n(𝝀)* necessarily limits the application to the visible regime, however, the benefit of using any conventional CCD as the detector expands the potential for real‐world applications using everyday camera systems. Changes in the light source or CCD do not affect the physics of the problem, and therefore any consistent setup should be able to achieve comparable results. Furthermore, the working wavelength region can be easily modified through the correct design of the metasurfaces in combination with the relative sensors, while any dielectric material with a high enough refractive index to produce the required Mie‐resonators could be employed for the colorimetric metasurfaces. Moreover, the requirement for the ultra‐precise nanofabrication of the meta‐atoms is also somewhat relaxed, as the exact reflected color of the metasurface is unimportant, as long as the fabrication conditions are kept consistent. With the implementation of large‐scale nanofabrication techniques such as nanoimprint lithography,^[^ ^44^ ^]^ larger metasurfaces could be employed to relax the restrictions of the micrometer‐scale metasurfaces used here, allowing for the collection of larger datasets in a single experiment. Finally, the integration of metasurfaces with a microfluidic channel opens up the potential for other tunable optical functionalities,^[^ ^45^ ^]^ while metasurfaces also possess the possibility of being utilized directly on‐chip for optical calculations.^[^ ^46^, ^47^ ^]^

In this work, we assumed that the liquid fully encapsulates the metasurface to modulate the color and spectral responses, which, along with the low viscosity of the oils, limits our ability to measure the thickness of the coatings. However, with well‐measured experimental data, the DNN could be extended further to provide an additional output value for the thickness or other desired parameters. Our proof‐of‐concept demonstration of glucose concentration measurements could also be extended to other interesting and useful transparent liquids in biological systems, such as blood serum and saline solutions. Furthermore, expanding this system to the measurement of the full complex refractive index of materials, including both the real and imaginary parts, has incredible potential in a wide range of fields. Potential methods to achieve this could be to exploit the Kramers‐Kronig relations alongside our system's ability to retrieve the real part of the refractive index across the visible spectrum or to train further DNNs with complex refractive index data. Additionally, by designing metasurfaces with chiral or anisotropic meta‐atoms, this platform could be expanded to chiral sensing or even the measurement of birefringence using a simple, easy‐to‐use, and inexpensive setup. With additional processing of data from multiple metasurfaces with resonances across the whole visible spectrum, we envision the potential to advance this system to identify unknown solutes, while the well‐trained models could be fine‐tuned.^[^ ^48^ ^]^ with additional data to measure the concentration of known substances in transparent liquids. Impurities that cause unwanted absorption peaks at certain wavelengths in otherwise clear liquids cause problems in measuring the refractive index of the pure solution through the reflected light, however, the flexibility of our system in terms of designing metasurfaces to reflect specific wavelengths of light and the ability to fine‐tune the model with a smaller dataset that includes impurities offer interesting research directions for future work. It should be noted that since we use a data‐driven AI technique, the precision of our system is inevitably limited by the precision of the original data.

Our system is extremely compact as it does not require any high‐cost optical components such as analyzers and requires no mechanically moving parts to conduct the measurements, components that are necessary to obtain Fresnel parameters using conventional ellipsometry. Moreover, it requires no additional intervention or calculation from an expert researcher to analyze the optical index dispersion of the target medium, which means the system can be easily implemented in workplaces without the need for extra specialist training. Our system also achieves high resolution over the entire visible spectrum, comparable to that of commercial Abbe refractometry which is limited to measure *n* at a single wavelength. Furthermore, we demonstrated a practical medical application of the system for the real‐time measurement of the concentration of glucose using a smartphone. This could also be potentially applied to the measurement of properties of various liquids in agriculture, liquor, or the sweetness of fruit. Improvements in the resolution of captured images could help to optimize the performance of our system further. This could be achieved with recent advancements in consumer smartphone optics systems, such as the macro imaging function in the iPhone 13 Pro, alongside low‐cost lighting attachments. Our refractive index measurement system pushes the boundaries of the integration of nanophotonics and AI that could influence the future of real‐life applications, particularly in the biomedical and sensing fields.

## 4 Experimental Section

### DNN Setup and Training

The DNNs were designed and written in Python using the Pytoch library. The DNN used to predict *n*_bg_ *(𝝀*) from reflectance spectra was made up of 4 hidden layers, with 300, 550, 900, and 500 neurons, respectively. The LeakyReLU activation function was used between all layers and trained using the ADAM optimizer with an initial learning rate of 1 × 10^−3^. and automatic reduction on plateau, until the model had converged. The DNNs for measuring *n*_bg_ *(𝝀)* from color images consisted of a convolutional neural network (CNN) with 3 2D convolution layers with a kernel size of 3, stride of 1, and padding of 1, with 6, 12, and 24 channels respectively. The CNN fed into a DNN made up of 3 hidden layers, with 1024, 512, and 256 neurons, with the output layer having 101 neurons, corresponding to a spectral resolution of *n*_bg_ *(𝝀)* of ≈4 nm. This was changed to a single neuron when used as the output for glucose concentration measurements. The L1 loss function was employed throughout, with a batch size of 10 for the spectral measurements, and 32 for the RGB color swatches. All DNNs were trained on a computer with 32 GB of RAM, using a NVIDIA GeForce GTX 1080 Ti GPU, and training took between several minutes (reflectance spectra), and tens of minutes (color swatches).

### Metasurface Fabrication

The metasurfaces were fabricated using commercial plasma‐enhanced chemical vapor deposition (BMR Technology, HiDep‐SC), inductively coupled plasma reactive‐ion etching, electron‐beam deposition (KVT, ENS‐4004), and electron‐beam lithography (Elionix, ELS‐7800, acceleration voltage of 100 kV). First, cleaned fused silica substrates were prepared, and then low‐loss hydrogenated amorphous silicon (a‐Si:H) was deposited using plasma‐enhanced chemical vapor deposition. A photoresist (Kayaku advanced materials, PMMA 950 A6) was spin‐coated on the deposited low loss a‐Si:H at 3,000 rpm for 1 min, before baking the fused silica substrate at 180 °C for 5 min. A conductive polymer (Showa Denko, Espacer 300Z) was spin‐coated at 2,000 rpm for 1 min to prevent electron accumulation during the electron‐beam lithography. Circle arrays were patterned with electron‐beam lithography, and then the exposed area was developed with the developer (Kayaku advanced materials, MIBK:IPA = 1:3) at 0 °C for 10 min. Chromium (Cr) mask was deposited onto the developed patterns using electron‐beam deposition before lift‐off of the undeveloped PMMA layer, creating circular Cr mask patterns that were transferred to low‐loss a‐Si:H patterns using inductively coupled plasma reactive‐ion etching. Finally, the low‐loss a‐Si:H metasurfaces were created after Cr‐mask etching by immersing them in Cr etchant (Transene, CE‐905N).

### Measurement of Reflectance Spectra

The reflectance spectra were measured with a homemade setup, which consists of a white light source (Newport, xenon lamp) and a spectrometer (HORIBA, iHR320). An objective lens with a numerical aperture of 0.4 was used to capture the microscopy images of the metasurfaces.

### Measurement of Reflected Colors Using a Microscope

The reflected colors from the metasurfaces were captured using an MX63 Industrial Microscope (Olympus, Japan). The halogen light source was illuminated using a TH4‐200 power supply (Olympus, Japan). An MY10X‐803 (Olympus, Japan) objective lens was used, and an Olympus C‐Mount 0.63X Camera Adapter (Olympus, Japan) was employed as the eyepiece. The exposure time was set to 100 ms.

### Micro‐Channel Fabrication

The microfluidic chip was designed with a width of 2,000 *µm* and a height of 500 *µm*. It was fabricated by cutting thin polymethylmethacrylate (PMMA) plates (500 *µm*‐thick; AcrylChoiga, South Korea) with a laser cutter (Machine shop, ML‐7050A, South Korea). 150 *µm*‐thick transparent glass (Scilab, Cov1031, South Korea) was used to make the ceiling for the microfluidic chip.

### Micro‐Channel Integration

The metasurfaces were integrated onto the bottom surface of the fabricated microfluidic chip using double‐sided adhesive (3 m, 300LSE, USA). A peristaltic pump (BQ80S+DW10‐2‐CE‐W, USA) generated the flow inside the microfluidic channel to expose the metasurfaces to various liquids, including the refractive index liquids, solutions of distilled water and glucose (1 kg, G8270, South Korea), and pure distilled water to flush and clean the channel.

### Measurement of Reflected Colors Using a Smartphone

A Galaxy Note 20 5G (Samsung, SM‐N981N) smartphone was mounted at a distance of ≈19.5 cm from the metasurfaces integrated into the microfluidic channel. At this distance, the smartphone was able to capture images of the metasurfaces without the need for any additional optics. Care was taken to ensure that the camera settings, such as the ISO (320), shutter speed (1/250 s), and white balance (5500 K) were constant throughout the experiments.

## Conflict of Interest

The authors declare no conflict of interest.

## Supporting information

Supporting Information

Supplemental Video 1

## Data Availability

The data that support the findings of this study are available from the corresponding author upon reasonable request.
