# Supplementary material for: Artificial Intelligence‐Enhanced Metasurfaces for Instantaneous Measurements of Dispersive Refractive Index
Source: Adv Sci (Weinh). 2024 Sep 3;11(39):2403143. doi: 10.1002/advs.202403143 (PMC11497055; doi:10.1002/advs.202403143)
Supplement: Supplementary file 1 — Supporting Information [file ADVS-11-2403143-s001.docx]

Supporting Information

Artificial Intelligence-Enhanced Metasurfaces for Instantaneous Measurements of Dispersive Refractive Index

*Trevon Badloe^1,2†^, Younghwan Yang^3†^, Seokho Lee^3†^, Dongmin Jeon^3^, Jaeseung Youn^3^, Dong* *Sung Kim^3^, and Junsuk Rho^3,4,5,6,7*^*

*^1^Graduate School of Artificial Intelligence, Pohang University of Science and Technology (POSTECH), Pohang, 37673 Republic of Korea*

*^2^Department of Electronics and Information Engineering, Korea University, Sejong 30019, Republic of Korea*

*^3^Department of Mechanical Engineering, Pohang University of Science and Technology (POSTECH), Pohang 37673, Republic of Korea*

*^4^Department of Chemical Engineering, Pohang University of Science and Technology (POSTECH), Pohang 37673, Republic of Korea*

*^5^Department of Electrical Engineering, Pohang University of Science and Technology (POSTECH), Pohang 37673, Republic of Korea*

*^6^POSCO-POSTECH-RIST Convergence Research Center for Flat Optics and Metaphotonics, Pohang 37673, Republic of Korea*

*^7^National Institute of Nanomaterials Technology (NINT), Pohang 37673, Republic of Korea*

**Corresponding author. E-mail: jsrho@postech.ac.kr*

*^†^These authors contributed equally to this work*

**Supplementary Note 1: Comparison of colorimetric metasurfaces and AI with other refractive index measurement systems**

The proposed refractive index (*n*) sensing system is compared with a conventional Abbe refractometer (Mettler toledo, Refractive index Cell RX4), and ellipsometry (Metricon, 2010/M) in **Table S1**. Our proposed system instantaneously measures *n* of fluid by analyzing colorimetric changes from microchannel-integrated metasurfaces. In the experiment using microfluidic channel integrated metasurfaces, the liquid takes around 8 seconds to fully encapsulate the metasurface. This time could be decreased by increasing the liquid flow rate. Compared to ellipsometry which requires substantial human-expert intervention, the trained DNN does not need any additional human interactions after measurement, such as optical dispersion modelling, and initial figures for iteration. Our system is also able to instantaneously detect *n(𝝀)* without any expensive components such as half-wave plates or linear polarizers, which are essential components to obtain the Fresnel parameters in ellipsometry. The resolution of our system is high enough to be applied in biomolecular sensing,^[S1]^ and could also be potentially increased further through additional training data that could be acquired using an automated measuring setup.

**Table S1. Comparison of refractive index sensing methods**

|  | **Ours** | **Abbe refractometry**^[S2]^ | **Ellipsometry**^[S2]^ |
| --- | --- | --- | --- |
| **Real-time measurement** | O | X | X |
| **Required expertise** | Low | Low | High |
| **Cost** | Low | Low | High |
| **Number of wavelengths** | Entire visible spectrum | Single | Single |
| **Refractive index resolution** | 1×10^-4^ | 1×10^-4^ | 1×10^-4^ |

Alongside these conventional measurement techniques, recent advancements in nanotechnology have given rise to new methodologies. Notably, scanning near-field optical microscopy (s-SNOM) and the use of surface plasmon resonances (SPR) are two prominent techniques. We compare them in **Table S2**. s-SNOM is an advanced technique used to analyze the optical properties of materials with nanoscale resolution.^[S3]^ The local refractive index and absorption characteristics of a sample are measured by analyzing the scattered light produced at the tip of a high-frequency probe. Although real-time measurement is not feasible due to data collection during AFM oscillation, and it cannot measure multiple wavelengths simultaneously, it offers significant advantages in terms of spatial resolution. SPR can be used to detect changes in the refractive index of a medium in contact with a metal surface.^[S4]^ The resolution is extremely high due to the sensitivity of SPR and can be used for real-time detection of changes in refractive index. The drawbacks, however, are that the refractive index change must be measured at the surface of the sample and is therefore not suitable for measurements across a large area.

**Table S2. Comparison with recent refractive index sensing methods**

|  | **Ours** | **s-SNOM**^[S3]^ | **SPR**^[S4]^ |
| --- | --- | --- | --- |
| **Real-time measurement** | O | X | O |
| **Required expertise** | Low | High | Medium |
| **Cost** | Low | High | Medium |
| **Number of wavelengths** | Entire visible spectrum | Single | Single |
| **Refractive index**  **resolution** | 1×10^-4^ | 1×10^-4^ ~ 1×10^-5^ | 1×10^-6^ |

**Supplementary Note 2: Design and simulated color palette of a-Si:H metasurfaces**


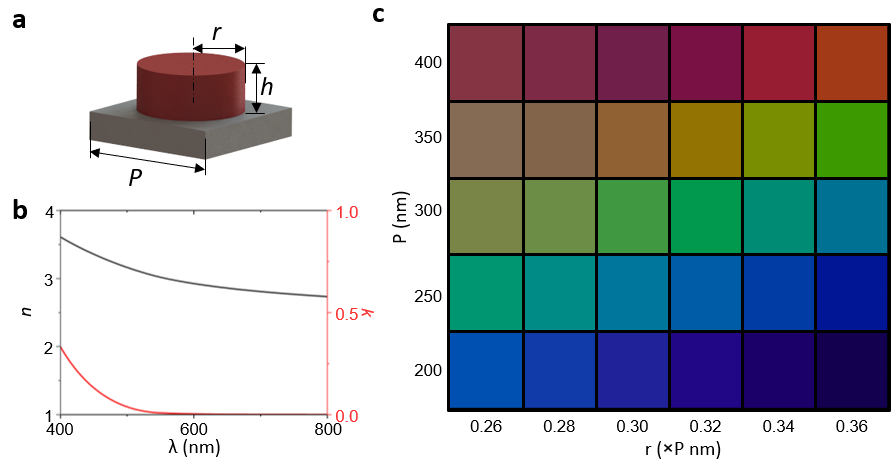


**Figure S1: The design and simulated color palette of the metasurfaces**. (a) Schematic illustration of the meta-atom that makes up the metasurfaces, a low-loss a-Si:H structure (red) with a radius (*r*), height (*h*), and periodicity (*P*), on top of a silica substrate (grey). (b) The measured refractive index of the low-loss a-Si:H. (c) The simulated color palette of the metasurfaces.

With a fixed meta-atom made of low-loss a-Si:H with height (*h*) of 110 nm in the background of air, a large spectrum of colors can be produced by varying the periodicity (*P*) and radius (*r*). A range of spectral colors and brightness are available to create data to train the DNN (Figure S1).

**Supplementary Note 3: Effect of *n_bg_* on reflected color**

Numerous research about structural color metasurfaces using dielectric materials has shown that the reflected color can be manipulated by changing the makeup of the meta-atom, substrate, or *n_bg_*.^[S5–S10]^ Changing the meta-atom structure or materials affects the inherent scattering properties that are seen through Mie-resonances,^[S10]^ while *n_bg_* also changes the refractive index contrast,^[S5,S9]^ and therefore the scattering properties. Furthermore, when the meta-atoms are arranged in a periodic array, they create a subwavelength 2D grating where the incident light is diffracted.^[S11]^ At the Rayleigh anomaly the diffracted light propagates at 90° to the meta-atoms that constitute the subwavelength grating. Quasi-guided mode resonances (qGMR) are induced when the grating provides the required momentum to the incident light for evanescent wavevectors to match the in-plane lattice propagation vectors, which are determined by *P*, and mode numbers *m* and *n* which define the diffraction orders in the *x*- and *y*-directions. When *n_bg_* is manipulated, the hybridization of all of the induced resonances, including the Mie-resonances and qGMR, is modulated in an unintuitive manner, leading to different reflectance across the wavelength region of interest.

**Supplementary Note 4: Measured reflectance spectra of metasurfaces**

**
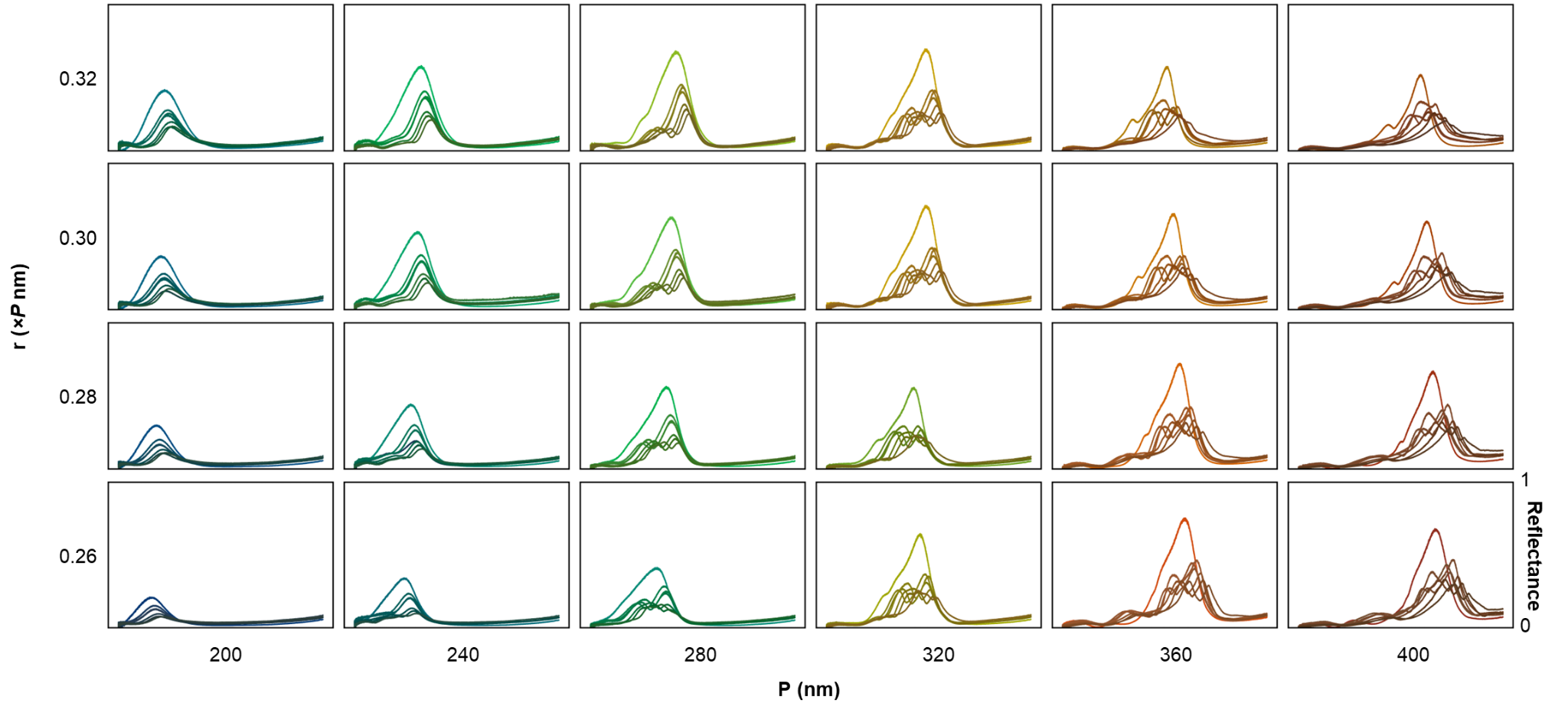
**

**Figure S2: Measured reflectance spectra of metasurfaces**. Each plot shows the measured reflectance spectra of each metasurface with the given geometric parameters in air and when coated. The color of the line represents the calculated color from the spectrum. The *x*-axes range from 400-800 nm.

When the metasurfaces are coated with a liquid, the measured reflectance spectra are heavily modified in a non-intuitive manner (Figure S2). Although distinct spectra are measured for each liquid, it would be impossible for a human to recover *n_bg_(𝝀)* from this spectral information alone*.*

**Supplementary Note 5: Variation in reflectance spectra of metasurfaces**


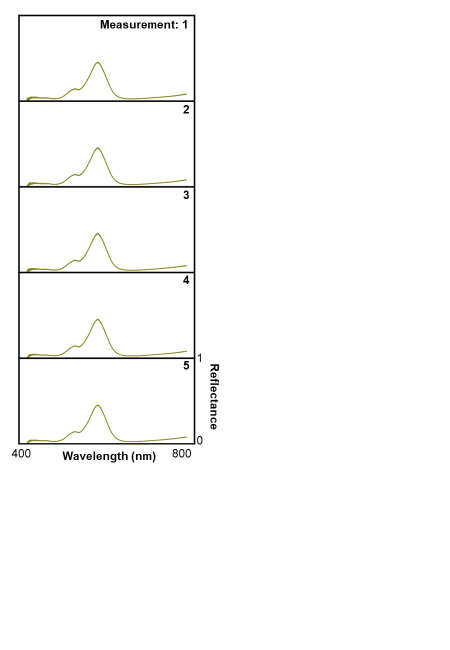


**Figure S3: Variation in measured reflectance spectra of the same metasurface**. Each plot is a separate measurement of the reflectance from the metasurface with *P* = 260 nm and *r* = 0.36*P* after coating, cleaning, and recoating with liquid number 1.

It is possible for the measured reflectance spectra to show slight variations due to differences in the measurement location that could have unwanted defects, or other undesired factors. Here, the measurements are fairly consistent for each of the 5 coating and cleaning cycles (Figure S3). Slight variations in the measured spectra could also be beneficial for training the DNN, as it adds a form of variation that could help with robustness.

##

**Supplementary Note 6: Optical microscope images of the metasurfaces**


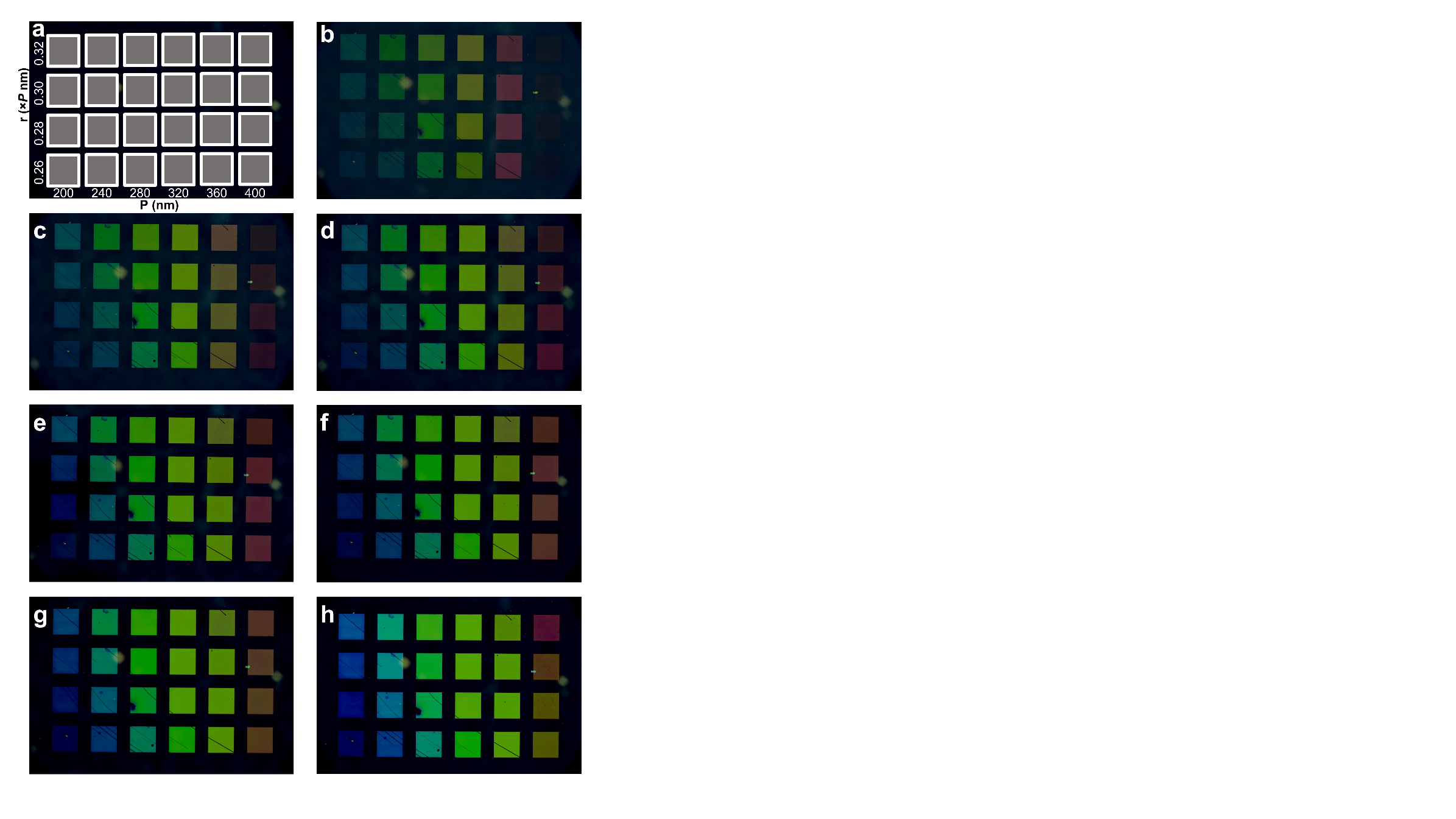


**Figure S4: Optical microscope images of the metasurfaces with different coatings**. (a) The layout of the geometric parameters of the metasurfaces. (b) Optical microscope image of the metasurfaces (b-g) coated with liquid 6-1 and (h) in air, respectively.

**Supplementary Note 7: Experimental setup of smartphone**


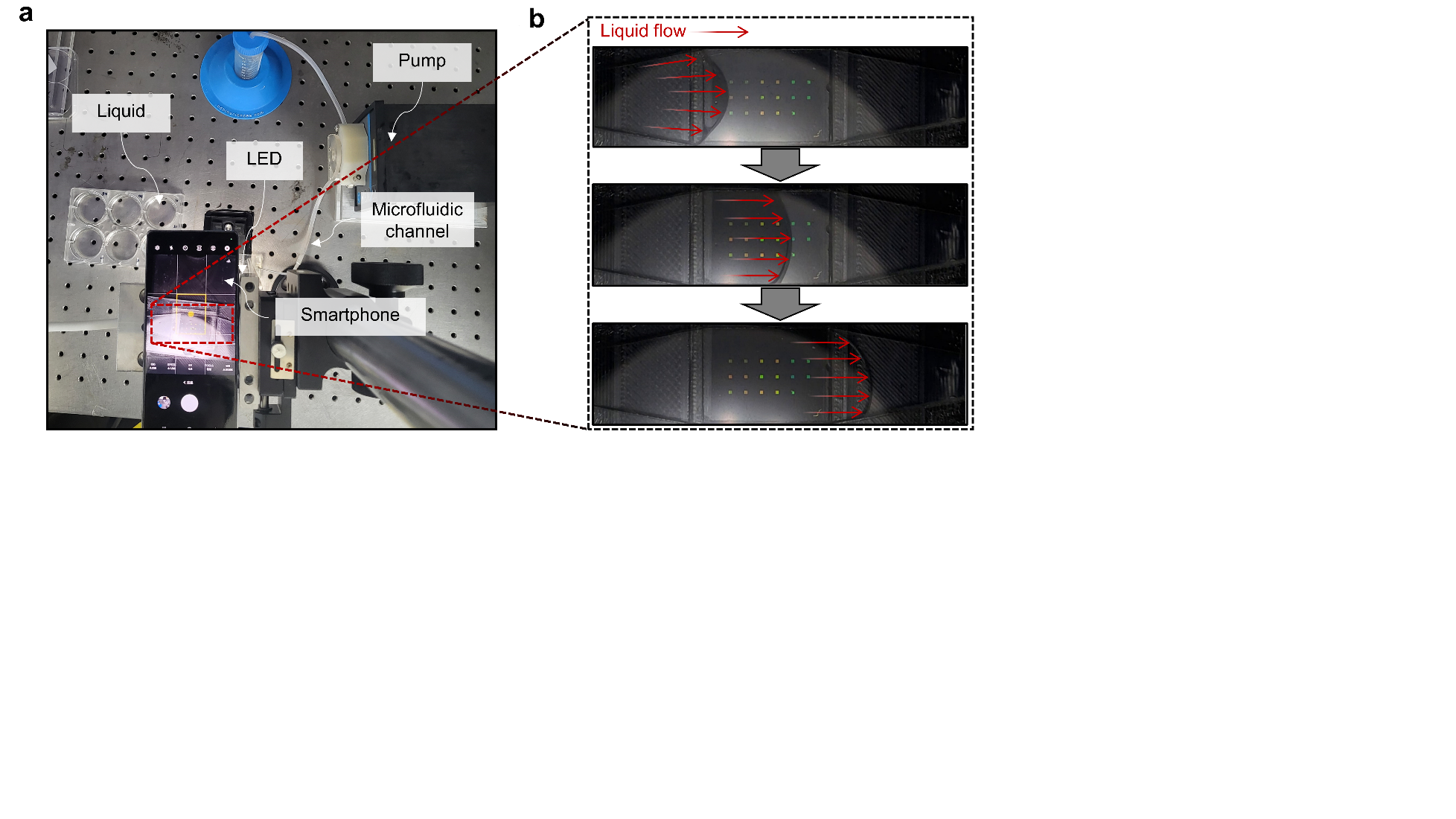


**Figure S5: Experimental results of the real-time measurement of refractive index from the RGB channels of a smartphone image.** (a) Experimental setup of the metasurface integrated microfluidic channel system. The LED illuminates the metasurfaces and videos are taken using the smartphone. (b) The liquids infiltrate the metasurfaces as they are pumped through the microfluidic channel, causing the reflected color to change.

**References**

S1. Y. Xu, P. Bai, X. Zhou, Y. Akimov, C. E. Png, L. Ang, W. Knoll, L. Wu, *Adv. Opt. Mater.* **2019**, *7*, 1801433.

S2. M. Ohring. *Materials Science of Thin Films: Deposition and Structure*. (Elsevier, 2001).

S3. D. E. Tranca, S. G. Stanciu, R. Hristu, B. M. Witgen, G. A. Stanciu, *Nanomedicine Nanotechnology, Biol. Med.* **2018**, *14*, 47.

S4. M. Piliarik, J. Homola, *Opt. Express* **2009**, *17*, 16505.

S5. J. Jang, T. Badloe, Y. Yang, T. Lee, J. Mun, J. Rho, *ACS Nano* **2020**, *14*, 15317.

S6. S. Sun, Z. Zhou, C. Zhang, Y. Gao, Z. Duan, S. Xiao, Q. Song, *ACS Nano* **2017**, *11*, 4445.

S7. B. Yang, W. Liu, Z. Li, H. Cheng, D. Y. Choi, S. Chen, J. Tian, *Nano Lett.* **2019**, *19*, 4221.

S8. Z. Dong, J. Ho, Y. F. Yu, Y. H. Fu, A. I. Kuznetsov, J. K. W. W. Yang, R. Paniagua-Dominguez, S. Wang, A. I. Kuznetsov, J. K. W. W. Yang, *Nano Lett.* **2017**, *17*, 7620.

S9. W. Yang, S. Xiao, Q. Song, Y. Liu, Y. Wu, S. Wang, J. Yu, J. Han, D.-P. Tsai, *Nat. Commun.* **2020**, *11*, 1864.

S10. T. Badloe, J. Kim, I. Kim, W.-S. Kim, W. S. Kim, Y.-K. Kim, J. Rho, *Light Sci. Appl.* **2022**, *11*, 118.

S11. Y. H. Ko, R. Magnusson, *Optica* **2018**, *5*, 289.
